# Further Proceedings of the Society

**Published:** 1841-09

**Authors:** 


### ARTICLE VI.

### FURTHER PROCEEDINGS OF THE SOCIETY.

The following gentlemen were elected members of the Society, in accordance with the provisions of the constitution and by-laws:

Wm. H. Burr, of Philadelphia; S. Dillingham, of Philadelphia; James Alcock, of New York; Charles Rowell, of New York; Geo. Washington Parmly, of New Orleans; David Roudolph Parmly, of Mobile; E. J. Dunning, of Buffalo, N. Y.; Chas. B. Foster, of Philadelphia; James Oscar Baldwin, of Newark, N. J.; William Ware, of Wilmington, N. C.; Frederick Crocker, of Sagharbour, L. I.; J. W. Shepherd, of Petersburg, Va.; James W. Cowan, of Dover, N. H.; Robt. McGrath, M. D. of Philadelphia; John C. McCabe, of Petersburg, Va.; Doctor Fisk, of Salem, Mass.; John Allen, of Cincinnati; G. G. Brewster, of Portsmouth, N. H.; T. B. Hamlin, of Wytheville, Va., B. B. Brown, M. D. of St. Louis, Mo.; John N. Frink, of Portland, Me.; Joseph Taylor, of Maysville, Ky.; Alexander Nelson, M. D. of Albany, N. Y.; Robert Nelson, of Albany, N. Y.; C. W. Grant, of Newburg; Doctor Macklin, of Vicksburg, Miss.; Benjamin Strickland, of Cleveland, Ohio; G. McDonald, M. D. of Macon, Ga.; J. P. Hullihen, of Wheeling, Va.; Wm. A. Ward, of Pittsburg, Pa.; R. Covington Mackall, D. D. S., Baltimore; Robert Arthur, D. D. S., Chambersburg, Pa.

### OFFICERS OF THE SOCIETY FOR THE YEARS 1841–42.

H. H. HAYDEN, M. D., *President.*
ELEAZAR PARMLY, M. D., *1st Vice President.*
LEWIS ROPER, M. D., *2d Vice President.*
SOLYMAN BROWN, M. D., *Recording Secretary.*
CHAPIN A. HARRIS, M. D., *Corresponding Secretary.*
ELISHA BAKER, M. D., *Treasurer.*
J. H. FOSTER, M. D., *Librarian.*

*Executive Committee.*

E. Townsend, E. Maynard,

L. S. Parmly, E. Taylor,

P. Houston.

*Examining Committee.*

H. M. Hayden, J. S. Gunnell,

J. McIlhenney, C. A. Harris,

J. Smith Dodge.

*Publishing Committee.*

H. S. Burr, E. Baker,

S. Brown, S. M. Shepherd,

S. W. Parmly.

Eleazar Parmly, M. D., was appointed to deliver the opening address at the next annual meeting, and the following gentlemen to deliver dissertations on subjects connected with Dental Theory or Practice, in accordance with a requisition of the by-laws of the Society: E. Baker, E. Townsend, V. Cuylor, H. M. Hayden, and E. Maynard.

J. H. Foster was appointed to prepare a popular essay on the importance of stopping teeth; E. Maynard, on that of the removal of salivary calculus from the teeth; H. H. Hayden, on the diseases of the gums; E. Townsend, on the importance of extracting diseased teeth, which cannot be restored; C. A. Harris, on first dentition.

We also extract from the proceedings of the Society the following, in relation to the establishment of a periodical:

*Resolved,* That this Society establish a periodical as the organ of its opinions on subjects of dental theory and practice, and for the purpose of advancing the interests of the science and the profession.

Thereupon, Dr. Harris, in the name of his coadjutors, made a formal tender of the American Journal of Dental Science, on conditions, that the present volume shall be completed by the original conductors, and also, that none of the engagements of the publishing committee of the first volume shall be violated by their successors, nor any of their contracts omitted.

This offer having been accepted, and the future plan of the publication determined on, C. A. Harris and Solyman Brown, were elected editors, and the following gentlemen, collaborators for the ensuing year, namely: H. H. Hayden, Eleazar Parmly, E. Maynard, H. M. Hayden, Elisha Townsend, Vernon Cuylor, Isaac J. Greenwood, G. G. Brewster, Ludolph Parmly, Edward Taylor, C. S. Brewster, Leonard Koecker, Lewis Roper, H. S. Burr, E. Baker, Horatio N. Fenn, Alexander Nelson, B. A. Rodrigues, Dr. Rogers, and Enoch Noyes.

The committee, Drs. E. Parmly, E. Baker, S. Brown, C. A. Harris, and J. Parmly, to whom the duty of reporting on the use of "lithodeon," "mineral paste," and all other substances of which mercury is an ingredient for stopping teeth, reported in substance, that the use of all such articles were hurtful, both to the teeth and every part of the mouth, and that there was no tooth, in which caries in it could be arrested, and the organ rendered serviceable by being filled, in which gold could not be employed. The report was unanimously adopted by the Society.

After a session of three days the Society adjourned to the third Tuesday in July, A. D. 1842, to meet at the TREMONT HOUSE, in BOSTON, at ten o'clock, unless otherwise ordered by the President.

